# Retrospective Analysis of the Efficacy of Integrated Lifestyle Modifications in Managing Prediabetes in the Indian Population

**DOI:** 10.1155/jdr/6172692

**Published:** 2025-08-13

**Authors:** Pramod Tripathi, Diptika Tiwari, Nidhi Kadam, Anagha Vyawahare, Baby Sharma, Thejas Kathrikolly, Malhar Ganla, Banshi Saboo

**Affiliations:** ^1^Department of Research, Freedom From Diabetes Research Foundation, Pune, Maharashtra, India; ^2^Department of Management, Freedom From Diabetes, Pune, Maharashtra, India; ^3^Department of Medicine, Dia Care-Diabetes Care and Hormone Clinic, Ahmedabad, Gujarat, India

**Keywords:** BMI, exercise, lifestyle intervention, plant-based diet, prediabetes remission, psychological support, sex, weight loss

## Abstract

**Background:** Prediabetes is a growing health concern in India, with a prevalence of 15.3%. This retrospective study was aimed at assessing the effectiveness of an integrated intensive lifestyle intervention (ILI) in Indian patients with prediabetes while exploring sex- and body mass index (BMI)–based differences.

**Methods:** This retrospective study analyzed data from 427 patients with prediabetes (HbA1c: 5.7%–6.4% not on insulin or oral hypoglycemic agents and aged 18–75 years) who underwent a 1-year online ILI program at the Freedom from Diabetes Clinic, India, between 2020 and 2023. The intervention consisted of a personalized plant-based diet, physical activity, psychological support, and medical management. Anthropometric and biochemical data were extracted from the clinical database, and logistic regression was used to identify factors associated with prediabetes remission.

**Results:** At the end of the study, 47.1% of patients achieved prediabetes remission. Significant weight loss and improvements were observed in HbA1c, BMI, fasting blood glucose, fasting insulin, insulin resistance, lipid profile, and blood pressure (*p* < 0.05). Notably, 34% of patients lost > 10% of their body weight. Females achieved greater reductions in weight and BMI than males (*p* < 0.001). The BMI stratified analysis revealed higher remission rates in the obesity group (BMI ≥ 25 kg/m^2^) (49.9%) than in the nonobesity group (BMI 18–24.9 kg/m^2^) (37.0%) (*p* = 0.028). The obesity group showed greater improvements in glycemic parameters and insulin resistance than the nonobesity group (*p* < 0.05). Logistic regression identified age ≤ 50 years (OR: 2.15, 95% CI: 1.42–3.27), endline HOMA-IR < 2.5 (OR: 1.60, 95% CI: 1.01–2.45), and > 10% weight loss (OR: 3.72, 95% CI: 2.34–5.90) as significant factors associated with prediabetes remission.

**Conclusions:** Prediabetes remission was achieved in almost half of the patients, highlighting the effectiveness of a tailored multidisciplinary approach for managing prediabetes in the Indian population. The study showed significant weight loss, improved glycemic control, and better lipid profiles and emphasized the need for personalized strategies for prediabetes management considering factors such as sex and BMI.

**Trial Registration:** Clinical Trials Registry of India identifier: CTRI/2024/03/064596

## 1. Introduction

Prediabetes refers to a state of nondiabetic hyperglycemia or intermediate glycemic conditions in individuals with glucose concentrations above healthy levels, but who do not meet the diagnostic criteria for diabetes [1]. This condition is characterized by abnormal carbohydrate metabolism [1] and increases the risk of developing Type 2 diabetes (T2D). Individuals with prediabetes also have a heightened risk of cardiovascular disease (CVD) and other diabetes-related complications [2].

Globally, prediabetes affects approximately 7.5% of adults aged 20–79 years (374 million individuals). If left untreated, prediabetes progresses to T2D at an estimated annual rate of 5%–10% [3, 4]. This issue is particularly pressing in India, where the prevalence of prediabetes is 15.3%. Additionally, more than 60% of these cases are expected to progress to diabetes within the next 4 years [5, 6]. This alarming trajectory is driven by a combination of factors, including the genetic predisposition of South Asians to insulin resistance, rapid urbanization, shifting dietary patterns, and an increasingly sedentary lifestyle [7]. The significant economic and healthcare burdens of prediabetes underscore the urgent need for effective diabetes prevention policies and interventions tailored to India's diverse sociocultural contexts [8, 9].

Unlike diabetes, prediabetes offers a crucial window of opportunity for intervention. By restoring healthy glucose concentrations, the onset of T2D and its complications can be delayed or prevented [10]. Prediabetes remission, defined as maintaining HbA1c below 39 mmol/mol (5.7%) without the use of any pharmacotherapy for at least 3 months [11], is influenced by factors such as sex and body mass index (BMI). Research suggests that males often experience more significant glycemic improvements and weight reductions than females [12]. Additionally, individuals with a lower BMI have higher remission rates than those with a higher BMI [13]. However, studies on prediabetes management using nonpharmacological and nonsurgical approaches, especially in the context of sex and BMI in the Indian population, are limited.

Further, research has indicated that lifestyle interventions can effectively lead to prediabetes remission and halt the progression of diabetes [14–16]. Despite the known benefits of lifestyle interventions in managing prediabetes, comprehensive studies that consider the unique dietary habits, genetic predispositions, and lifestyle factors of the Indian population are lacking. Existing research has often focused on isolated aspects of lifestyle interventions, such as diet or exercise; thus, a comprehensive framework that considers the interconnected nature of lifestyle factors affecting prediabetes management is needed [17–20]. Therefore, it is crucial to address this gap and understand how a multidisciplinary integrated intensive lifestyle intervention (ILI) can be optimized to prevent progression to diabetes in the Indian population, leading to more effective and culturally adaptable intervention strategies.

Given the variability and distinct characteristics of the Indian population, this retrospective study was aimed at evaluating the effectiveness of a tailored ILI program for managing prediabetes, with a focus on remission and exploration of sex- and BMI-related differences. As an exploratory analysis, the study assessed real-world trends in metabolic outcomes from a routinely implemented intervention, rather than testing predefined hypotheses in a controlled trial setting. Additionally, we aimed to identify factors associated with prediabetes remission to inform the development of more personalized and effective early intervention strategies for the Indian population.

## 2. Materials and Methods

### 2.1. Study Design, Settings, and Patients

**Figure 1:**
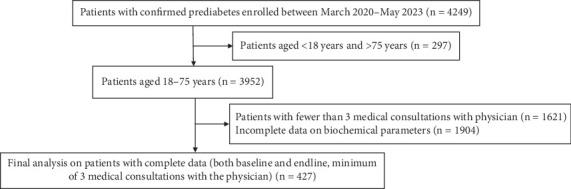
Flowchart for the selection of the patients for the study.

This retrospective study used data from the electronic records of the Freedom from Diabetes Clinic database in Pune, India. The clinic delivers a subscription-based online lifestyle intervention program as part of the routine care for prediabetes, diabetes, and obesity in a real-world setting. Data were extracted from 427 patients with prediabetes who were enrolled in the 1-year online integrated ILI program between March 2020 and May 2023 from 89 cities in India who met the eligibility criteria. Prediabetes was defined according to the American Diabetes Association (ADA) guidelines as HbA1c between 39 and 47 mmol/mol (5.7%–6.4%) [11]. Inclusion criteria were as follows: at baseline, patients aged 18–75 years with HbA1c concentration between 39 and 47 mmol/mol (5.7%–6.4%); not taking oral hypoglycemic agents or insulin (drug-naïve); without chronic conditions such as heart disease, diabetic neuropathy, liver disease, or renal disorders; who completed the 1-year program; and had complete data from at least three medical consultations during the intervention. The patient selection process is shown in Figure 1.

### 2.2. Intervention

**Figure 2:**
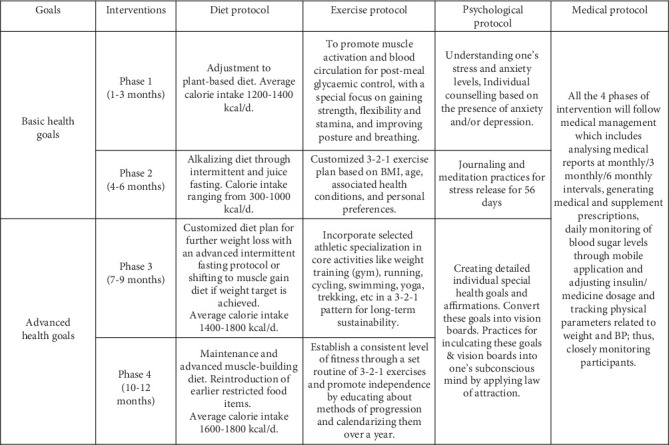
Timeline and components of interventions [21].

The 1-year online integrated ILI consisted of four phases, focusing on four core protocols: dietary modification, physical activity, psychological support, and medical management. The detailed protocol has been previously described [21]. Each patient was assigned a team of six experts: physician, dietician, physical therapist, psychologist, mentor (a past participant in the program), and monitor (for follow-up and consultation scheduling). At baseline, the core team of experts (dietitian, physical therapist, and physician) set basic health goals for each patient: the physician set the targets for HbA1c, the dietitian set goals for weight loss and BMI, and the physical therapist set the target for exercises based on pre-existing conditions. These basic health goals were set for the first 6 months and revisited at 6 months to set advanced health goals for the last 6 months of the 1-year intervention (Figure 2).

The dietary intervention included a plant-based diet (lentil-based recipes, sprouts, and vegetable salads) tailored to patients' BMI. The first phase focused on a balanced alkaline diet rich in vegetables, antioxidants, and phytonutrients, with a calorie intake of approximately 1200–1400 kcal/day. In the second phase, 1 month of progressive intermittent fasting and juice fasting was recommended for patients with a BMI over 23 kg/m^2^, reducing calorie intake to 300–500 kcal on juice-fasting days to achieve basic health goals with respect to BMI. The detailed protocol for progressive intermittent fasting has been previously described [21]. Intermittent fasting was not recommended for patients with normal to low BMI (< 23 kg/m^2^). Once the target BMI was achieved according to the basic health goals, calorie intake was gradually increased to 1400–1600 kcal/day, with a protein intake of 1–1.2 g/kg body weight to support muscle building with the help of exercises. The final phase focused on maintaining good glycemic control with a calorie intake of 1600–1800 kcal/day and increased protein intake based on the exercise plan under the supervision of a dietician and physical therapist. This phase aimed to help individuals manage their diets, sustain their exercise regimens, and maintain weight loss.

The exercise intervention was aimed at building and sustaining strength, flexibility, and stamina. Phase 1 focused on improving blood circulation, muscle activation, and antigravity exercises, such as warm-up, *Sooryanamaskaras* (sun salutations), super brain yoga [22], and palm plank exercises to improve postmeal glycemic control. Phase 2 emphasized muscle gain, weight loss, and core strengthening. Phase 3 provided personalized exercise plans (e.g., swimming, running, cycling, and yoga) based on the patient's BMI, comorbidities, age, and preferences for athletic specializations. The final phase implemented periodized exercise plans to sustain fitness levels in terms of strength, stamina, and flexibility, which were achieved during the intervention.

The third psychological support protocol aimed to enhance patients' understanding of stress, anxiety, and mind–body connections through journaling and meditation (group sessions). It also included individual counseling sessions by trained psychologists (upon request by patients) using techniques such as cognitive behavior therapy (CBT), rational emotive behavior therapy (REBT), neurolinguistic programming (NLP), clinical hypnotherapy, life coaching, and pranic healing [23, 24].

Lastly, medical management involved the assessment of patients' health using biochemical tests and adjustment of medication doses based on the self-reported blood glucose concentration in a mobile application, along with addressing micronutrient deficiency through supplements prescribed by the assigned physician. Pre-existing comorbidities, including dyslipidemia and hypertension, were also assessed. In addition to daily dosage adjustments, the physician conducted a minimum of three medical consultations with each participant over the course of the 1-year program.

As a prerequisite, all patients had a digital weighing scale, sphygmomanometer, and glucometer for monitoring and self-reporting weight, blood pressure, and blood glucose concentration, respectively.

### 2.3. Mode of Delivery

The intervention included access to a dedicated mobile application for patients. The intervention was delivered online and included video conferences and telephone calls, with an average of 63 calls per patient per year. The mobile application facilitated communication, vital sign monitoring, and access to region-specific plant-based recipes, along with prerecorded exercise videos tailored to specific physical conditions and audio recordings of meditations. This comprehensive approach ensured that diet, physical activity, and stress management plans were personalized for each patient, supported by extensive resources including exercise videos, meditation audio, and customized recipes. Throughout the intervention, patients received counseling and motivational support to encourage adherence to the protocol. Adherence to the program was monitored through a mobile application supported by weekly and fortnightly consultations with dietitians and physical therapists, which transitioned to monthly sessions to maintain engagement. Physician consultations were scheduled quarterly, and self-reported blood glucose concentrations were monitored daily via a mobile application. Patients could also connect with their respective care teams through the application via text messages and calls. They were also encouraged to attend monthly online group sessions for education and motivation to enhance their adherence.

### 2.4. Measurement of Anthropometric and Biochemical Parameters

Data on anthropometric measurements (height and weight), biochemical parameters (HbA1c, fasting blood glucose [FBG], postprandial blood glucose [PP-BG], fasting insulin, and lipid profile), and medical history (prediabetes duration, comorbidities, and medication status) of all eligible patients were extracted from the database for both baseline (pre-intervention) and endline (post-1-year intervention). To ensure the accuracy of self-reported anthropometric measurements, the program provided comprehensive guidelines on the necessary precautions for measuring weight and height (patients were instructed to measure and report the average of three weight readings). BMI was computed based on these measurements. The homeostatic model assessment of insulin resistance (HOMA-IR), homeostatic model assessment of beta-cell function (HOMA-B), and quantitative insulin sensitivity check index (QUICKI) at both baseline and the end of 1 year were calculated using standard formulas [25, 26]. Non-high-density lipoprotein cholesterol (non-HDL-C) concentration was calculated from the lipid profile using the following formula (non‐HDL‐C = total cholesterol − HDL‐C).

### 2.5. Statistical Analysis

Statistical analyses were performed using IBM SPSS software (Version 21.0). The normality of the data distribution was assessed using the Kolmogorov–Smirnov test. Continuous variables are presented as medians (interquartile range [IQR]) owing to the skewed distributions of all variables. For categorical variables, data are presented as frequencies and percentages. To evaluate intervention-related changes, the percentage change from baseline to endline was calculated for each participant. The absolute percentage change was calculated as the difference between the final and initial values divided by the initial value and then multiplied by 100. Group comparisons were performed using a linear mixed-effects model (LMM) to account for repeated measures and to adjust for relevant covariates. This approach addressed potential baseline imbalances and interaction effects, thereby enhancing the interpretability of findings. Estimates and confidence intervals are reported up to two decimal places unless greater precision was necessary. Wilcoxon signed-rank and McNemar's tests were used to compare the baseline and endline data. The chi-square test was used to test the association between categorical variables. Binary logistic regression analysis was used to assess factors associated with prediabetes remission. Significant factors in the bivariate analysis were included in the binary logistic regression model (age, baseline BMI, endline HOMA-IR, endline non-HDL cholesterol, and percentage weight loss at the endline). Additionally, a sensitivity analysis was performed on 264 patients who were not on statins to check the reliability of the lipid profile findings. Given the exploratory nature of this study and its focus on real-world outcomes, no formal adjustments for multiple comparisons were made. The results were interpreted with caution, recognizing the increased risk of Type I errors associated with multiple analyses. Statistical significance was set at *p* < 0.05.

## 3. Results

### 3.1. Baseline Characteristics of the Study Population

Data were available for 427 patients with prediabetes.  Table 1 shows the baseline characteristics of the study participants. The mean age of the patients was 50.9 ± 9.8 years, the median duration of prediabetes was 2.5 years (IQR, 1.5–3.9), and 60% (*n* = 256) of the patients were female. Most of the patients were married (89%, *n* = 380) and had a family history of diabetes (62.8%, *n* = 268). At baseline, 38.2% (*n* = 163) and 30.4% (*n* = 130) of patients were on statins or other lipid-lowering medications and antihypertensive medications, respectively. At baseline, 78.5% (*n* = 335) had obesity (BMI ≥ 25 kg/m^2^) according to the WHO Asia-Pacific Guidelines [27].

### 3.2. Improvement in HbA1c, Anthropometric, and Biochemical Parameter Postintervention

At endline, without adjustment, HbA1c significantly improved (*p* < 0.001). Of the total patients, 47.1% (*n* = 201) achieved prediabetes remission, 1.2% (*n* = 5) progressed to diabetes (HbA1c ≥ 6.5%), and 52.9% (*n* = 221) remained in the prediabetes range (HbA1c 5.7%–6.4%). Significant improvements were also observed in weight, BMI, fasting insulin, FBG, HOMA-IR, QUICKI, lipid profile, and blood pressure (all *p* < 0.05; Table 1). However, HOMA-B significantly decreased at endline (*p* < 0.001).

Thirty-four percent of the patients (*n* = 144) had a weight loss of > 10%. At baseline, 78.5% of the patients had a BMI of ≥ 25 kg/m^2^, indicating obesity; at endline, this percentage decreased to 60%. Additionally, the number of patients taking antihypertensive medication reduced from 30.4% to 25.7% at the endline (*p* < 0.001).

A sensitivity analysis was performed, excluding participants who used statins or other lipid-lowering medications during the 12-month intervention, to ensure that the observed changes in lipid parameters were attributable to the lifestyle intervention itself rather than pharmacological effects (Supporting Information 1: Table S1). The results showed that the outcomes remained consistent for total cholesterol, triglyceride, LDL-C, and non-HDL-C, indicating the impact of the intervention on lipid profile. For HDL-C, while the change in prevalence did not reach statistical significance in the medication-free subgroup, the odds ratio analysis showed a trend toward improvement, with the odds of HDL-C increase being 1.81 in females and 2.22 in males, postintervention. These findings suggest a modest benefit of the intervention on HDL-C, even in the absence of medication use, with the reduced significance likely due to the smaller sample size and lower statistical power (Supporting Information 1: Table S1). The prevalence of other lipid parameters, such as total cholesterol, triglyceride, LDL-C, and non-HDL-C, continued to show consistent results between the total cohort and those who did not take any lipid-lowering medications, further supporting the effectiveness of the lifestyle intervention (Supporting Information 1: Table S1).

Of the 427 patients with prediabetes, 38.2% (*N* = 163) were taking medications for dyslipidemia management at baseline. Of these, medications were tapered to a complete stoppage in 27.1% (*n* = 44) of patients while maintaining lipid concentration within healthy limits. Additionally, statistically significant improvements were noted in the lipid profile: high total cholesterol (≥ 5.17 mmol/L) decreased from 37.0% to 28.1% (*p* = 0.001), high triglycerides (≥ 1.7 mmol/L) decreased from 29.5% to 20.4% (*p* ≤ 0.001), high non-HDL (≥ 3.36 mmol/L) decreased from 61.4% to 52.9% (*p* = 0.001), and low HDL-C decreased from 46.8% to 34.5% (*p* = 0.002) (male ≤ 1.02 mmol/L) and 64.1% to 54.7% (*p* = 0.007) (female ≤ 1.3 mmol/L) at the end of the intervention. However, no significant improvement was observed in LDL-C (≥ 2.6 mmol/L), which decreased from 71.2% to 70.3% (*p* = 0.764).

Furthermore, we assessed sex- and BMI-wise differences in the effectiveness of the intervention.

### 3.3. Sex-Wise Comparison of Anthropometric and Biochemical Parameters

Table 2 summarizes the changes in anthropometric and biochemical parameters across sex groups and evaluates the interaction effects between time and sex. Significant differences between males and females were observed in weight, BMI, FBG, systolic blood pressure, total cholesterol, and HDL-C (*p* < 0.05). Postintervention, prediabetes remission was achieved in 49.6% of females and 43.3% of males (*p* = 0.199).

From baseline to endline, both sexes showed significant improvements in weight, BMI, HbA1c, fasting insulin, FBG, HOMA-IR, QUICKI, systolic and diastolic blood pressure, triglycerides, non-HDL, and HDL-C (all *p* < 0.05). However, no significant changes were observed in HOMA-B (*p* = 0.129), total cholesterol (*p* = 0.296), or LDL-C (*p* = 0.349).

Interaction analysis showed that females had slightly greater reductions in weight (*p* = 0.014) and BMI (*p* < 0.001), but smaller reductions in systolic BP (*p* = 0.022) compared to males. Changes in glycemic outcomes (HbA1c, FBG, and fasting insulin), insulin resistance/sensitivity markers (HOMA-IR, HOMA-B, and QUICKI), diastolic blood pressure, and lipid parameters (total cholesterol, triglycerides, LDL-C, non-HDL-C, and HDL-C) were similar between the sexes (*p* > 0.05).

Among the covariates, older age was significantly associated with lower weight, BMI, fasting insulin, HOMA-B, and triglyceride concentrations (all *p* < 0.05) but slightly higher HbA1c (*p* = 0.005) and HDL-C concentrations (*p* < 0.001). Age showed no significant association with FBG, HOMA-IR, QUICKI, systolic or diastolic BP, total cholesterol, LDL-C, or non-HDL-C.

Additionally, HOMA-IR was positively associated with HOMA-B (*p* < 0.001), and HOMA-B was inversely associated with QUICKI (*p* < 0.001). Patients not on antihypertensive and lipid-lowering medications significantly had lower blood pressure and lower concentrations of total cholesterol, triglycerides, LDL-C, and non-HDL-C (all *p* < 0.001). No differences with medications were observed for HDL-C (*p* = 0.882).

### 3.4. BMI-Wise Comparison of Anthropometric and Biochemical Parameters

Table 3 presents a comparison of anthropometric and biochemical improvements across the BMI groups and examines the interaction effects between time and BMI classification. Participants with obesity had a higher rate of prediabetes remission (49.9%) than those in the nonobesity group (37.0%) (*p* = 0.028). Significant differences in weight, BMI, HbA1c, fasting insulin, FBG, HOMA-B, total cholesterol, LDL-C, and non-HDL-C concentrations were observed between the obesity and nonobesity groups (*p* < 0.05). Additionally, significant baseline-to-endline improvements were observed in both BMI groups for weight, BMI, HbA1c, fasting insulin, FBG, HOMA-IR, QUICKI, systolic and diastolic blood pressure, triglyceride, non-HDL, and HDL-C concentrations (all *p* < 0.05). However, changes in HOMA-B (*p* = 0.052), total cholesterol (*p* = 0.150), and LDL-C (*p* = 0.115) were not statistically significant.

Interaction analysis (BMI × time) revealed that the obesity group experienced significantly greater reductions in weight (*p* < 0.001), BMI (*p* < 0.001), fasting insulin (*p* = 0.002), FBG (*p* = 0.023), and HOMA-IR (*p* = 0.001), along with improved insulin sensitivity (QUICKI, *p* < 0.001), compared to the nonobesity group. The changes in HOMA-B, blood pressure (systolic and diastolic), and lipid parameters (total cholesterol, triglycerides, LDL-C, non-HDL-C, and HDL-C) did not differ significantly between the groups (*p* > 0.05).

Regarding covariates, older age was significantly associated with lower weight, BMI, fasting insulin, HOMA-B, total cholesterol, and triglyceride concentrations (all *p* < 0.05) and slightly higher HbA1c (*p* = 0.003) and HDL-C concentrations (*p* < 0.001). No significant associations were observed between age and FBG, HOMA-IR, QUICKI, blood pressure (systolic or diastolic), LDL-C, or non-HDL-C concentrations.

As expected, HOMA-IR was positively associated with HOMA-B (*p* < 0.001), whereas HOMA-B was inversely associated with QUICKI (*p* < 0.001), reinforcing their interconnected role in glycemic physiology. Patients not on antihypertensive and lipid-lowering medications significantly had lower blood pressure and lower concentrations of total cholesterol, triglycerides, LDL-C, and non-HDL-C (all *p* < 0.001) but showed no significant difference for HDL-C (*p* = 0.778). Additionally, female sex had significantly higher HDL-C concentrations (*p* < 0.001).

### 3.5. Factors Associated With Prediabetes Remission

**Figure 3:**
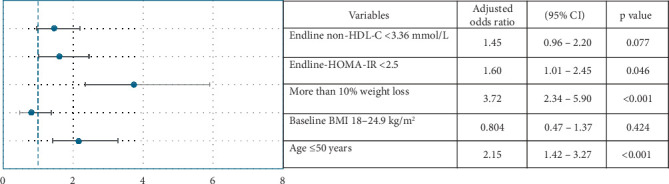
Significant factors associated with prediabetes remission identified through logistic regression. Footnote: the forest plot displays adjusted odds ratios (AORs) with 95% confidence intervals (CIs) for various parameters to identify associated factors. Each circle represents the point estimate, and the horizontal lines show the 95% confidence intervals, which reflect the range within which the true AOR falls. The dashed vertical line at AOR = 1 represents the line of no effect for variables.

Bivariate analysis revealed that baseline characteristics such as age under 50 years and BMI ≥ 25 kg/m^2^ were significantly associated with prediabetes remission (*p* < 0.05). Additionally, postintervention measures including HOMA-IR (< 2.5), non-HDL cholesterol (< 3.36 mmol/L), and weight loss greater than 10% were also significantly associated with remission (*p* < 0.05). To further explore these relationships, we conducted a binary logistic regression analysis, which identified age under 50, endline HOMA-IR < 2.5, and > 10% weight loss as significant factors associated with achieving remission (Figure 3).

## 4. Discussion

Prediabetes represents a crucial stage in the continuum of metabolic health, offering an opportunity for preventive interventions to mitigate the progression of T2D and its complications [10]. This study showed that an ILI incorporating a plant-based diet, physical activity, psychological support, and medical management led to remission in 47.1% of patients. Unlike previous studies that focused on individual interventions [17–20], this comprehensive approach improved HbA1c, weight, BMI, FBG concentration, insulin resistance, lipid profile, and blood pressure. Females achieved greater weight and BMI reductions, whereas individuals with obesity showed higher remission rates. Key factors associated with remission included younger age (≤ 50 years), lower endline insulin resistance (HOMA-IR < 2.5), and > 10% weight loss.

A significant outcome was the successful remission of prediabetes in 47.1% of patients after a 1-year lifestyle intervention, exceeding the 44% remission rate noted in a previous Canadian study using a multidomain lifestyle intervention consisting of expert-guided educational and nutritional counseling combined with personalized physical exercise [28]. This aligns with the existing evidence on the potential of lifestyle interventions to prevent or delay T2D onset in prediabetic individuals [20, 29, 30]. Another notable finding was substantial weight loss, with 34% of patients losing > 10% of their initial body weight, surpassing the results reported by Sandforth et al. (5% weight loss in 27.5% of patients with prediabetes) [31].

Our study patients showed significant improvements in glycemic parameters, including HbA1c and FBG concentrations, showing a transition from prediabetes to normoglycemia, which is consistent with other studies reporting the effect of interventions, including dietary modification and physical activity, in reversing prediabetes and preventing T2D [18, 31, 32]. Improvements in insulin sensitivity, as evidenced by reductions in HOMA-IR and an increase in QUICKI, corroborate the findings of other lifestyle intervention studies [32–34]. These changes in insulin dynamics are crucial for preventing T2D progression and for reflecting the metabolic benefits of the intervention. We also observed significant improvements in lipid profiles, with 27.1% of the patients discontinuing medications for dyslipidemia management while maintaining healthy lipid profiles. This highlights the efficacy of customized ILI for dyslipidemia management in patients with prediabetes, which is consistent with previous studies [18, 35].

In the Indian context, limited information exists on sex- and BMI-based studies on prediabetes remission. Our study showed that both females and males achieved significant weight loss and improvements in other metabolic parameters, consistent with previous studies [36]. However, sex-by-time interaction analyses revealed that females achieved greater reductions in weight and BMI than males, which is in contrast with the PREVIEW study, which reported that females benefited less than males in weight loss and body mass composition with lifestyle intervention consisting of a low-energy diet and physical activity due to greater lean body mass and males having physiological advantages in losing weight under caloric restriction [37]. However, a possible explanation for our findings could be sex-based behavioral differences. Women are often found to be more consistent in adhering to dietary guidelines, more engaged with structured programs, and more likely to adopt supportive health behaviors during lifestyle interventions [38, 39]. These adherence-related advantages may offset the physiological disadvantages and contribute to the greater weight loss and reduction in BMI observed in the female participants in our study.

Unadjusted change in HOMA-IR and HOMA-B showed a significant decline from baseline to endline, as determined by Wilcoxon signed-rank tests. However, in the adjusted sex-wise LMM analysis, only HOMA-IR showed significant improvement, with a reduction in insulin resistance; no significant changes were observed for HOMA-B. Further, LMM analysis revealed no significant main effects of sex or sex-by-time interaction, suggesting that the effect of the intervention was similar across sexes. Percentage change analysis indicated a greater decline in HOMA-B among females, though this was not statistically significant after adjusting for repeated measures. HOMA-IR was positively correlated with HOMA-B in the LMM, reflecting a compensatory relationship between insulin resistance and insulin secretion. The observed trend toward a larger beta-cell decline in females may relate to sex-specific differences in fat distribution and hormonal influences [40, 41]. Females generally have more subcutaneous and less visceral fat, which supports better insulin sensitivity and reduced metabolic risk [38]. Estrogen may further enhance glucose regulation by promoting favorable fat distribution and improving insulin activity [38, 41]. These physiological differences could explain the greater HOMA-B decline in females with initial compensatory hypersecretion, followed by improved insulin sensitivity. Although not statistically significant, these consistent patterns underscore the relevance of biological sex in interpreting metabolic responses to lifestyle interventions.

Both obesity and nonobesity groups showed significant improvements from baseline to endline in weight, BMI, glycemic markers (HbA1c and FBG), fasting insulin, insulin resistance indices (HOMA-IR and QUICKI), blood pressure, and lipid parameters (triglycerides, non-HDL, and HDL-C). However, group-by-time interaction analysis revealed that the obesity group experienced significantly greater improvements in weight, BMI, FBG, fasting insulin, and insulin resistance markers (HOMA-IR and QUICKI). These findings are consistent with a study from China that reported higher rates of diabetes remission among obese compared to lean individuals [40]. The greater metabolic improvements observed in patients with obesity may have resulted from their more severe baseline metabolic dysfunction, including elevated insulin resistance, visceral adiposity, and systemic inflammation, which are more amenable to change through lifestyle interventions [41]. Conversely, lean individuals typically exhibit beta-cell dysfunction with normal insulin sensitivity, experiencing modest changes due to a ceiling effect that limits the extent of metabolic change [42].

Regarding beta-cell function (BCF), post-LMM BMI-wise analysis, which appropriately models repeated measures and adjusts for relevant covariates, HOMA-B showed a slight overall increase from baseline to endline, with marginal statistical significance. Higher absolute HOMA-B values were observed in the obesity group (BMI ≥ 25 kg/m^2^). Although the percentage decline in HOMA-B was more pronounced in the obesity group and a slight increase was seen in the nonobesity group (*BMI* < 25 kg/m^2^), the group × time interaction was not statistically significant, indicating similar trends across BMI categories. As seen in the sex-stratified model, the BMI-stratified analysis showed a significant positive association between HOMA-IR and HOMA-B, reflecting a compensatory increase in insulin secretion in response to insulin resistance. The greater decline in HOMA-B among patients with obesity is consistent with previous studies, suggesting that elevated HOMA-B at baseline may reflect compensatory hyperinsulinemia that diminishes as insulin sensitivity improves with weight loss through lifestyle intervention [43]. In contrast, the nonobese group showed a modest, nonsignificant increase in HOMA-B, likely reflecting enhanced beta-cell responsiveness rather than compensation, which may be due to defects in insulin secretion at baseline [44]. These patterns highlight how insulin secretion adapts to metabolic demands, emphasizing the need to interpret HOMA-B changes within the context of insulin resistance [45]. In our study, baseline obesity (BMI ≥ 25 kg/m^2^) was significantly associated with prediabetes remission, contradicting the findings of Han et al., who reported a lower remission rate in Chinese patients with a higher BMI [13]. This difference may be due to body composition variations between the Indian and Chinese populations, as the former show higher levels of visceral adiposity and insulin resistance at lower BMI values [44]. As mentioned earlier, patients with pre-existing obesity may have experienced more substantial metabolic improvements, including significant weight loss and improved insulin sensitivity, which are key drivers of glucose normalization and potentially result in higher remission rates [31].

Our results showed that age less than 50 years at baseline, HOMA-IR < 2.5 at endline, and weight loss > 10% at endline were significant factors associated with prediabetes remission. This aligns with previous research indicating that weight loss and reduced insulin resistance serve as key drivers of prediabetes remission, while younger age has also been identified as a significant factor contributing to remission outcomes [31, 43]. Younger adults generally exhibit lower levels of insulin resistance, as reflected by lower HOMA-IR values, which enhance glucose regulation and responsiveness to lifestyle interventions [46]. Moreover, HOMA-IR has been shown to increase with both chronological and biological aging, underscoring the role of advancing age in impairing insulin action and thus reducing the likelihood of prediabetes remission [46]. Sex-based physiological and behavioral factors may further explain the differential remission outcomes. For instance, males often present with greater insulin resistance and more central adiposity, which may affect their baseline metabolic risk profile, thus impacting remission status [47]. However, remission was not influenced by sex in the present study. Ethnicity-specific differences also appear to contribute to the observed heterogeneity in remission. For example, African Americans may experience more rapid beta-cell decline, while Asian Indians are predisposed to higher hepatic lipid accumulation and muscle insulin resistance, despite lower BMI, attributable to greater visceral and ectopic fat deposition [48, 49]. Such intrinsic differences could modulate both baseline risk and the metabolic response to interventions, thus influencing prediabetes remission.

Taken together, these results highlight the importance of personalized prediabetes management strategies that consider age-related metabolic activity, weight change, sex differences, and ethnicity-specific metabolic traits. Our study also highlights the importance of inclusive approaches that incorporate nutritional, physical, and mental well-being along with medical management for prediabetes in the Indian population. This aligns with an expert group consensus report that recognizes that holistic management extends beyond conventional interventions [6].

Despite its merits, this study had several limitations. The retrospective, single-center design may have introduced selection bias and limited the generalizability beyond the Indian population. The lack of randomization increases the risk of confounding, and reliance on self-reported online data may have affected the accuracy despite precautions taken during the data collection. Due to the holistic nature of the intervention, the effects of the individual components could not be isolated. Only participants who completed the program and had complete biochemical data were included, making this a per-protocol analysis. While informative for evaluating efficacy among engaged users, excluding noncompleters may have further biased the results. Owing to data limitations, fasting glucose and oral glucose tolerance test (oGTT) values were not consistently available, and we used HbA1c, an ADA-recommended marker, for diagnosing and tracking prediabetes. However, this may have excluded individuals with isolated impaired fasting glucose (IFG) or impaired glucose tolerance (IGT), potentially leading to under- or overestimation of remission. Given the known differences in IFG and IGT pathophysiology across populations, the absence of subclassification further limits cross-cohort comparisons. Future studies with detailed metabolic phenotyping and greater ethnic diversity are needed. Importantly, we did not phenotype the participants based on the underlying mechanisms of hyperglycemia. Differences such as hepatic versus muscle insulin resistance [50], gastric emptying [51], energy regulation [52], sex-based factors [47, 52], and ethnic-specific risks (e.g., beta-cell dysfunction in East Asians and insulin resistance in South Asians) [48, 49] were not assessed but may influence outcomes. Despite these limitations, our study, spanning 89 Indian cities, demonstrated the feasibility of delivering scalable, personalized lifestyle interventions for prediabetes management and prevention of its progression to diabetes.

In conclusion, our study demonstrates that tailored, multidisciplinary approaches and ILIs are effective in achieving prediabetes remission among Indian adults. The observed remission rates, along with significant improvements in weight and metabolic parameters, underscore the potential of ILI in reducing the burden of T2D. Additionally, sex- and BMI-specific outcomes highlight the importance of personalized approaches. Future multicenter studies are needed to assess the long-term sustainability and generalizability of these findings.

## Figures and Tables

**Table 1:** Changes in anthropometric and biochemical parameters post-1-year intervention (*N* = 427).

**Parameters**	**Baseline**	**Endline**	**Change at 12 months (IQR)**	**p** ** values**
Weight (kg)	77.0 (68.0 to 89.0)	71.0 (63.0 to 81.0)	−5.8 (−9.6 to −2.3)	< 0.001
BMI (kg/m^2^)	29.0 (25.6 to 33.3)	26.0 (23.5 to 30.1)	−2.1 (−3.6 to −0.81)	< 0.001
HbA1c (mmol/mol)	42.0 (40.9 to 44.2)	38.7 (35.5 to 40.9)	−3.8 (−6.5 to −1.0)	< 0.001
HbA1c (%)	6.0 (5.9 to 6.2)	5.7 (5.4 to 5.9)	−0.35 (−0.6 to −0.1)	
Fasting insulin (pmol/L)	65.4 (42.5 to 98.3)	48.8 (32.7 to 75.0)	−12.4 (−33.6 to 5.8)	< 0.001
Fasting blood glucose (mmol/L)	5.5 (5.1 to 6.1)	5.3 (4.9 to 5.7)	−0.27 (−0.68 to 0.16)	< 0.001
HOMA-B	110.0 (66.5 to 171.2)	95.3 (62.2 to 144.0)	−7.3 (−45.8 to 20.8)	< 0.001
HOMA-IR	2.7 (1.7 to 4.0)	2.0 (1.3 to 3.0)	−0.66 (−1.5 to 0.23)	< 0.001
QUICKI	0.32 (0.31 to 0.35)	0.34 (0.32 to 0.36)	0.014 (−0.006 to 0.032)	< 0.001
BP—Systolic (mm/Hg)	120.0 (115.0 to 130.0)	120.0 (114.0 to 123.0)	−1.0 (−10.0 to 0.10)	< 0.001
BP—Diastolic (mm/Hg)	80.0 (76.0 to 84.0)	80.0 (75.0 to 80.0)	0.0 (−6.0 to 0.0)	< 0.001
Total cholesterol (mmol/L)	4.8 (4.1 to 5.4)	4.6 (4.1 to 5.2)	−0.07 (−0.64 to 0.40)	0.005
Triglycerides (mmol/L)	1.4 (1.0 to 1.8)	1.1 (0.9 to 1.5)	−0.15 (−0.46 to 0.79)	< 0.001
LDL-C (mmol/L)	3.1 (2.5 to 3.6)	2.9 (2.4 to 3.4)	−0.10 (−0.56 to 0.31)	0.002
Non-HDL-C (mmol/L)	3.7 (3.0 to 4.2)	3.4 (2.8 to 4.0)	−0.15 (−0.67 to 0.32)	< 0.001
HDL-C (mmol/L)	1.1 (0.9 to 1.2)	1.2 (1.0 to 1.4)	0.07 (−0.05 to 0.20)	< 0.001

*Note:* Data are presented as median (interquartile range). Baseline and endline measurements were compared using the Wilcoxon signed-rank test. Non‐HDL‐C = total cholesterol − HDL‐C.

Abbreviations: BMI, body mass index; BP, blood pressure; HbA1c, glycated hemoglobin; HDL-C, high-density lipoprotein cholesterol; HOMA-B, homeostatic model assessment of beta cell function; HOMA-IR, homeostatic model assessment of insulin resistance; LDL-C, low-density lipoprotein cholesterol; non-HDL-C, non-high-density lipoprotein cholesterol; QUICK, Quantitative insulin sensitivity check index.

**Table 2:** Sex-wise comparison of anthropometric and biochemical parameters post-1-year intervention.

**Outcome**	**Group (sex)**	**Baseline**	**Endline**	**% change (IQR)**	**Model effect**	**Estimate (95% CI)**	**p** ** value**
Weight (kg)	Female	76.3 (67.5 to 88.0)	69.5 (60.8 to 79.2)	−8.1 (−12.1 to −5.2)	Time (endline vs. baseline)	−5.64 (−6.54 to −4.73)	**< 0.001**
	Male	78.6 (69.0 to 90.5)	74.0 (65.5 to 85.0)	−5.8 (−10.3 to −0.6)	Group (female vs. male)	−6.05 (−8.78 to −3.31)	**< 0.001**
					Group∗time	1.47 (0.30 to 2.64)	**0.014**
	Age	−0.39 (−0.52 to −0.25)	**< 0.001**

BMI (kg/m^2^)	Female	30.4 (26.7 to 34.4)	27.3 (24.2 to 31.2)	−8.1 (−12.1 to −5.4)	Time (endline vs. baseline)	−1.91 (−2.25 to −1.57)	**< 0.001**
	Male	26.7 (23.8 to 30.6)	25.0 (22.7 to 28.3)	−5.7 (−10.4 to −0.60)	Group (female vs. male)	2.04 (1.10 to 2.97)	**< 0.001**
					Group∗time	0.89 (0.45 to 1.33)	**< 0.001**
	Age	−0.12 (−0.16 to −0.07)	**< 0.001**

HbA1c (%)	Female	6.0 (5.8 to 6.1)	5.7 (5.4 to 5.8)	−5.2 (−9.8 to −3.1)	Time (endline vs. baseline)	−0.36 (−0.41 to −0.31)	**< 0.001**
	Male	6.1 (5.9 to 6.3)	5.7 (5.5 to 5.9)	−6.2 (−9.6 to −1.6)	Group (female vs. male)	−0.48 (−0.11 to 0.02)	0.145
					Group∗time	−0.009 (−0.07 to 0.05)	0.783
	Age	0.003 (−0.074 to 0.056)	**0.005**

Fasting insulin (pmol/L)	Female	65.7 (44.0 to 97.8)	48.6 (32.4 to 74.7)	−23.3 (−44.6 to 7.9)	Time (endline vs. baseline)	−13.14 (−19.21 to −7.07)	**< 0.001**
	Male	64.9 (39.9 to 98.6)	50.3 (33.7 to 75.9)	−19.4 (−39.6 to 17.6)	Group (female vs. male)	0.35 (−7.65 to 8.36)	0.931
					Group∗time	0.95 (−6.86 to 8.78)	0.241
	Age	−1.08 (−1.44 to −0.73)	**< 0.001**

Fasting blood glucose (mmol/L)	Female	5.4 (5.1 to 5.9)	5.2 (4.8 to 5.6)	−5.1 (−11.1 to 3.8)	Time (endline vs. baseline)	−0.33 (−0.44 to −0.21)	**< 0.001**
	Male	5.6 (5.1 to 6.3)	5.3 (5.0 to 5.8)	−4.7 (−12.0 to 2.6)	Group (female vs. male)	−0.16 (−0.31 to −0.02)	**0.022**
					Group∗time	−0.10 (−0.25 to 0.05)	0.184
	Age	0.003 (−0.002 to 0.010)	1.075

HOMA-B	Female	110.4 (71.5 to 164.7)	95.8 (63.4 to 148.6)	−11.4 (−36.4 to 19.1)	Time (endline vs. baseline)	7.22 (−2.11 to 16.57)	0.129
	Male	104.0 (58.0 to 176.5)	91.5 (56.8 to 138.6)	−5.0 (−34.3 to 35.9)	Group (female vs. male)	8.84 (−2.42 to 20.11)	0.124
					Group∗time	3.34 (−8.56 to 15.25)	0.552
	Age	−1.01 (−1.53 to −0.49)	**< 0.001**
	HOMA-IR	26.42 (24.18 to 28.66)	**< 0.001**

HOMA-IR	Female	2.7 (1.7 to 4.0)	1.8 (1.2 to 2.9)	−26.4 (−49.8 to 4.8)	Time (endline vs. baseline)	−0.50 (−0.72 to −0.28)	**< 0.001**
	Male	2.6 (1.7 to 3.9)	1.9 (1.3 to 2.9)	−24.7 (−47.2 to 17.0)	Group (female vs. male)	−0.17 (−0.44 to 0.09)	0.201
					Group∗time	−0.02 (−0.31 to 0.25)	0.850
	Age	−0.01 (−0.02 to 0.001)	0.089
	HOMA-B	0.014 (0.013 to 0.015)	**< 0.001**

QUICKI	Female	0.32 (0.31 to 0.35)	0.35 (0.32 to 0.36)	4.5 (−0.70 to 10.8)	Time (endline vs. baseline)	0.008 (0.003 to 0.012)	**< 0.001**
	Male	0.33 (0.31 to 0.35)	0.34 (0.32 to 0.36)	4.0 (−2.5 to 9.5)	Group (female vs. male)	0.002 (−0.002 to 0.007)	0.327
					Group∗time	−0.003 (−0.008 to 0.002)	0.248
	Age	0.0001 (−0.0006 to 0.0003)	0.152
	HOMA-B	−0.0002 (−0.0003 to 0.0002)	**< 0.001**

BP—Systolic (mm/Hg)	Female	120.0 (111.5 to 128.5)	120.0 (110.0 to 120.0)	0.0 (−7.6 to 1.9)	Time (endline vs. baseline)	−4.77 (−7.35 to −2.20)	**< 0.001**
	Male	121.5 (120.0 to 130.0)	120.0 (118.0 to 126.0)	0.0 (−9.0 to 0.85)	Group (female vs. male)	−2.39 (−4.38 to −0.41)	**0.018**
					Group∗time	−3.86 (−7.17 to −0.56)	**0.022**
	Age	0.06 (−0.03 to 0.16)	0.214
	Antihypertensive (no/yes)	−3.74 (−5.81 to −1.66)	**< 0.001**

BP—Diastolic (mm/Hg)	Female	80 (74.0 to 80.0)	80.0 (70.0 to 80.0)	0.0 (−6.8 to 0.00)	Time (endline vs. baseline)	−2.33 (−3.98 to −0.68)	**0.006**
	Male	80.0 (80.0 to 88.7)	80.0 (75.0 to 82.0)	0.0 (−9.0 to 0.85)	Group (female vs. male)	−0.84 (−2.49 to 0.80)	0.315
					Group∗time	−1.34 (−3.46 to 0.78)	0.215
	Age	−0.04 (−0.13 to 0.03)	0.258
	Antihypertensive (no/yes)	−3.68 (−5.33 to −2.02)	**< 0.001**

Total cholesterol (mmol/L)	Female	4.9 (4.2 to 5.5)	4.7 (4.2 to 5.3)	−1.7 (−11.2 to 8.9)	Time (endline vs. baseline)	−0.07 (−0.21 to 0.06)	0.296
	Male	4.6 (4.0 to 5.3)	4.5 (3.9 to 4.9)	−1.5 (−13.2 to 9.1)	Group (female vs. male)	0.29 (0.13 to 0.45)	**< 0.001**
					Group∗time	0.002 (−0.174 to 0.179)	0.978
	Age	0.007 (−0.0001 to 0.015)	0.054
	Lipid-lowering drugs (no/yes)	−0.41 (−0.56 to −0.27)	**< 0.001**

Triglycerides (mmol/L)	Female	1.4 (1.0 to 1.7)	1.1 (0.89 to 1.52)	−11.5 (−29.2 to 6.7)	Time (endline vs. baseline)	−0.21 (−0.30 to −0.13)	**< 0.001**
	Male	1.4 (1.1 to 1.9)	1.1 (0.92 to 1.6)	−12.0 (−33.6 to 7.8)	Group (female vs. male)	−0.03 (−0.13 to 0.07)	0.567
					Group∗time	−0.06 (−0.17 to 0.04)	0.245
	Age	−0.006 (−0.011 to −0.001)	**0.013**
	Lipid-lowering drugs (no/yes)	−0.36 (−0.46 to −0.26)	**< 0.001**

LDL-C (mmol/L)	Female	3.1 (2.6 to 3.6)	2.9 (2.5 to 3.5)	−3.2 (−15.6 to 11.6)	Time (endline vs. baseline)	−0.05 (−0.18 to 0.06)	0.349
	Male	2.9 (2.3 to 3.6)	2.7 (2.4 to 3.3)	−4.6 (−15.8 to 12.3)	Group (female vs. male)	0.13 (−0.01 to 0.28)	0.064
					Group∗time	0.03 (−0.12 to 0.19)	0.668
	Age	0.001 (−0.005 to 0.008)	0.624
	Lipid-lowering drugs (no/yes)	−0.29 (−0.42 to −0.16)	**< 0.001**

Non-HDL-C (mmol/L)	Female	3.7 (3.0 to 4.3)	3.5 (2.9 to 4.0)	−4.4 (−15.0 to 11.3)	Time (endline vs. baseline)	−0.16 (−0.30 to −0.02)	**0.018**
	Male	3.6 (2.8 to 4.2)	3.3 (2.8 to 3.8)	−6.2 (−17.9 to 9.6)	Group (female vs. male)	0.14 (−0.01 to 0.30)	0.073
					Group∗time	−0.01 (−0.18 to 0.16)	0.869
	Age	−0.004 (−0.007 to 0.006)	0.908
	Lipid-lowering drugs (no/yes)	−0.41 (−0.55 to −0.26)	**< 0.001**

HDL-C (mmol/L)	Female	1.1 (1.0 to 1.3)	1.3 (1.1 to 1.5)	5.0 (−4.5 to 17.5)	Time (endline vs. baseline)	0.09 (0.06 to 0.12)	**< 0.001**
	Male	1.0 (0.90 to 1.1)	1.1 (0.97 to 1.3)	7.8 (−2.0 to 20.8)	Group (female vs. male)	0.14 (0.10 to 0.19)	**< 0.001**
					Group∗time	0.017 (−0.020 to 0.054)	0.374
	Age	0.008 (0.006 to 0.010)	**< 0.001**
					Lipid-lowering drugs (no/yes)	−0.004 (−0.040 to 0.032)	0.822

*Note:* Baseline, endline, and percentage change data for various parameters for both female and male patients are presented as medians (interquartile range [IQR]). The model effect included time; group, including female and male; and their interaction with time and with the covariates (age, HOMA-IR, HOMA-B, antihypertensive, and lipid-lowering drugs). Data are presented as estimates with 95% CI, considering baseline (time point) as the reference category for change over time, considering male and endline (time point) as the reference categories for group sex and group time interaction, and considering “yes” as the reference category for antihypertensive and lipid-lowering drugs; estimates are shown to two or three decimal places based on variable scale and interpretability; % change represents the unadjusted median percentage change from baseline to endline within each group and is provided to enhance clinical interpretability. Model effects (estimate, 95% CI, and *p* value) reflect adjusted comparisons derived from the linear mixed model. *p* value: significance of difference between baseline and endline for the respective group; all the significant values are presented in bold.

Abbreviations: BMI, body mass index; BP, blood pressure; HbA1c, glycated haemoglobin; HDL-C, high-density lipoprotein cholesterol; HOMA-B, homeostatic model assessment of beta-cell function; HOMA-IR, homeostatic model assessment of insulin resistance; LDL-C, low-density lipoprotein cholesterol; QUICKI, quantitative insulin sensitivity check index.

**Table 3:** BMI-wise comparison of anthropometric and biochemical parameters post-1-year intervention.

**Outcome**	**Group (BMI)**	**Baseline**	**Endline**	**% change (IQR)**	**Model effect**	**Estimate (95% CI)**	**p** ** value**
Weight (kg)	Nonobesity	62.5 (56.2 to 67.9)	59.7 (55.5 to 64.5)	−3.0 (−6.5 to 0.0)	Time (endline vs. baseline)	−7.68 (−8.29 to −7.08)	**< 0.001**
	Obesity	81.8 (73.9 to 92.0)	74 (66.5 to 85.6)	−8.5 (−12.8 to −5.2)	Group (nonobesity vs. obesity)	−15.62 (−18.63 to 12.60)	**< 0.001**
					Group∗time	−5.39 (−6.70 to −4.08)	**< 0.001**
	Age	−0.34 (−0.46 to −0.21)	**< 0.001**

BMI (kg/m^2^)	Nonobesity	22.9 (21.8 to 23.9)	22.1 (21.0 to 23.3)	−2.4 (−6.5 to 0.71)	Time (endline vs. baseline)	−2.92 (−3.15 to −2.69)	**< 0.001**
	Obesity	30.6 (27.7 to 34.4)	27.7 (25.0 to 31.4)	−8.6 (−12 to −5.2)	Group (nonobesity vs. obesity)	−6.42 (−7.39 to −5.45)	**< 0.001**
					Group∗time	−2.17 (−2.66 to −1.67)	**< 0.001**
	Age	−0.08 (−0.12 to −0.04)	**< 0.001**

HbA1c (%)	Nonobesity	6.1 (5.9 to 6.3)	5.8 (5.5 to 6.0)	−5.1 (−8.4 to −1.5)	Time (endline vs. baseline)	−0.37 (−0.41 to −0.34)	**< 0.001**
	Obesity	6.0 (5.9 to 6.2)	5.7 (5.4 to 5.8)	−5.7 (−10.3 to −3.1)	Group (nonobesity vs. obesity)	0.12 (0.04 to 0.20)	**0.001**
					Group∗time	−0.069 (−0.147 to 0.007)	0.077
	Age	0.003 (0.001 to 0.005)	**0.003**

Fasting insulin (pmol/L)	Nonobesity	42.4 (27.0 to 59.8)	40.9 (25.6 to 54.9)	−5.0 (−35.0 to 39.7)	Time (endline vs. baseline)	−16.88 (−21.16 to −12.6)	**< 0.001**
	Obesity	73.8 (49.8 to 107.7)	55.2 (35.0 to 82.5)	−25.4 (−45.8 to 7.1)	Group (nonobesity vs. obesity)	−19.91 (−29.29 to −10.53)	**< 0.001**
					Group∗time	−14.60 (−23.80 to −5.41)	**0.002**
	Age	−0.948 (−1.29 to −0.60)	**< 0.001**

Fasting blood glucose (mmol/L)	Nonobesity	5.6 (5.1 to 6.2)	5.4 (5.0 to 5.9)	−3.2 (−9.1 to 5.7)	Time (endline vs. baseline)	−0.31 (−0.39 to −0.23)	**< 0.001**
	Obesity	5.5 (5.1 to 6.1)	5.2 (4.8 to 5.6)	−5.2 (−12.0 to −2.4)	Group (nonobesity vs. obesity)	0.26 (0.09 to 0.43)	**0.002**
					Group∗time	−0.20 (−0.38 to −0.02	**0.023**
	Age	0.002 (−0.005 to 0.008)	0.575

HOMA-B	Nonobesity	62.2 (40.1 to 109.1)	64.1 (45.4 to 95.2)	4.5 (−35.6 to 51.6)	Time (endline vs. baseline)	6.76 (−0.67 to 13.59)	0.052
	Obesity	124.4 (76.2 to 186.0)	109.0 (68.6 to 159.8)	−11.1 (−35.5 to 17.3)	Group (nonobesity vs. obesity)	−27.90 (−41.25 to −14.55)	**< 0.001**
					Group∗time	9.09 (−5.12 to 23.31)	0.209
	Age	−0.87 (−1.39 to −0.36)	**0.001**
	HOMA-IR	25.76 (23.49 to 28.03)	**< 0.001**

HOMA-IR	Nonobesity	1.8 (1.0 to 2.3)	1.6 (1.0 to 2.3)	−18.1 (−38.5 to 29.1)	Time (endline vs. baseline)	−0.61 (−0.77 to −0.46)	**< 0.001**
	Obesity	3.0 (2.0 to 4.6)	2.1 (1.3 to 3.2)	−27.9 (−50.3 to 2.5)	Group (nonobesity vs. obesity)	−0.63 (−0.39 to 0.26)	0.703
					Group∗time	−0.57 (−0.91 to −0.24)	**0.001**
	Age	−0.010 (−0.022 to 0.002)	0.099
	HOMA-B	0.014 (0.0130 to 0.015)	**< 0.001**

QUICKI	Nonobesity	0.35 (0.33 to 0.38)	0.35 (0.33 to 0.38)	2.2 (−4.7 to 7.2)	Time (endline vs. baseline)	0.013 (0.010 to 0.016)	**< 0.001**
	Obesity	0.32 (0.30 to 0.34)	0.34 (0.32 to 0.36)	4.7 (−0.39 to 11.0)	Group (nonobesity vs. obesity)	0.005 (−0.001 to 0.011)	0.129
					Group∗time	0.0138 (0.007 to 0.020)	**< 0.001**
	Age	0.0001 (−0.00009 to 0.0004)	0.240
	HOMA-B	−0.00022 (−0.0003 to −0.00020)	**< 0.001**

BP—Systolic (mm/Hg)	Nonobesity	120.0 (115.0 to 130.0)	120.0 (113.0 to 122.0)	0.0 (−6.9 to 1.7)	Time (endline vs. baseline)	−2.67 (−4.54 to −0.80)	**0.005**
	Obesity	120.0 (116.3 to 130.0)	120.0 (114.0 to 123.5)	−1.6 (−7.6 to 0.0)	Group (nonobesity vs. obesity)	−0.32 (−2.62 to 1.98)	0.784
					Group∗time	−1.03 (−4.84 to 2.77)	0.595
	Age	0.06 (−0.04 to 0.16)	0.257
	Antihypertensive (no/yes)	−4.07 (−6.15 to −1.98)	**< 0.001**

BP—Diastolic (mm/Hg)	Nonobesity	80.0 (70.0 to 81.5)	80.0 (70.0 to 80.0)	0.0 (−6.4 to 1.3)	Time (endline vs. baseline)	−1.80 (−3.00 to −0.61)	**0.003**
	Obesity	80 (77.0 to 85.0)	80.0 (75.2 to 80.0)	0.0 (−8.4 to 0.0)	Group (nonobesity vs. obesity)	−1.87 (−3.76 to 0.02)	0.053
					Group∗time	−1.20 (−3.63 to 1.22)	0.329
	Age	−0.36 (−0.12 to 0.04)	0.395
	Antihypertensive (no/yes)	−3.79 (−5.43 to 2.15)	**< 0.001**

Total cholesterol (mmol/L)	Nonobesity	4.5 (4.0 to 5.3)	4.4 (3.8 to 4.9)	−0.28 (−11.9 to 8.8)	Time (endline vs. baseline)	−0.07 (−0.17 to 0.02)	0.150
	Obesity	4.8 (4.1 to 5.4)	4.7 (4.1 to 5.3)	−1.8 (−12.0 to 9.0)	Group (nonobesity vs. obesity)	−0.27 (−0.46 to −0.07)	**0.007**
					Group∗time	0.03 (−0.17 to 0.24)	0.770
	Age	0.010 (0.002 to 0.018)	**0.012**
	Lipid-lowering drugs (no/yes)	−0.37 (−0.52 to −0.22)	**< 0.001**

Triglycerides (mmol/L)	Nonobesity	1.2 (0.90 to 1.5)	1.1 (0.85 to 1.4)	−6.5 (−28.6 to 14.1)	Time (endline vs. baseline)	−0.20 (−0.25 to −0.12)	**< 0.001**
	Obesity	1.4 (1.1 to 1.8)	1.2 (0.91 to 1.6)	−12.7 (−31.8 to 5.7)	Group (nonobesity vs. obesity)	−0.09 (−0.22 to 0.03)	0.147
					Group∗time	−0.05 (−0.19 to 0.07)	0.395
	Age	−0.006 (−0.011 to −0.0008)	**0.023**
	Lipid-lowering drugs (no/yes)	−0.36 (−0.46 to −0.26)	**< 0.001**

LDL-C (mmol/L)	Nonobesity	2.9 (2.3 to 3.5)	2.7 (2.1 to 3.2)	−3.7 (−17.0 to 12.2)	Time (endline vs. baseline)	−0.07 (−0.15 to 0.01)	0.115
	Obesity	3.1 (2.5 to 3.6)	2.9 (2.5 to 3.5)	−3.6 (−15.7 to 11.7)	Group (nonobesity vs. obesity)	−0.21 (−0.39 to −0.03)	**0.017**
					Group∗time	0.05 (−0.13 to 0.23)	0.585
	Age	0.003 (−0.003 to 0.010)	0.334
	Lipid-lowering drugs (no/yes)	−0.26 (−0.40 to 0.13)	**< 0.001**

Non-HDL-C (mmol/L)	Nonobesity	3.4 (2.8 to 4.1)	3.2 (2.7 to 3.7)	−3.8 (−16.7 to 9.6)	Time (endline vs. baseline)	−0.15 (−0.24 to −0.05)	**0.002**
	Obesity	3.7 (2.9 to 4.3)	3.5 (2.9 to 4.0)	−5.3 (−16.3 to 11.1)	Group (nonobesity vs. obesity)	−0.25 (−0.44 to −0.06)	**0.008**
					Group∗time	0.03 (−0.17 to 0.24)	0.726
	Age	0.002 (−0.006 to 0.009)	0.690
	Lipid-lowering drugs (no/yes)	−0.38 (−0.52 to −0.24)	**< 0.001**

HDL-C (mmol/L)	Nonobesity (female)	1.2 (1.1 to 1.6)	1.4 (1.2 to 1.6)	4.0 (−4.0 to 16.0)	Time (endline vs. baseline)	0.08 (0.06 to 0.10)	**< 0.001**
	Nonobesity (male)	0.99 (0.85 to 1.1)	1.1 (0.94 to 1.2)	8.9 (−1.0 to 18.8)	Group (nonobesity vs. obesity)	−0.02 (−0.03 to 0.08)	0.626
	Obesity (female)	1.1 (1.0 to 1.3)	1.2 (1.1 to 1.4)	5.5 (−4.7 to 17.8)	Group∗time	−0.006 (−0.050 to 0.040)	0.797
	Obesity (male)	1.0 (0.94 to 1.2)	1.1 (0.98 to 1.3)	7.3 (−2.3 to 21.4)	Age	0.008 (0.006 to 0.010)	**< 0.001**
					Lipid-lowering drugs (no/yes)	−0.005 (−0.041 to 0.031)	0.778
					Sex (females/males)	0.16 (0.12 to 0.20)	**< 0.001**

*Note:* Baseline, endline, and percentage change data on various parameters for both nonobesity and obesity patients are presented as medians (interquartile range [IQR]). The model effect included time, group including nonobesity and obesity, and their interaction with time and with covariates (age, HOMA-IR, HOMA-B, antihypertensive, and lipid-lowering drugs). Data are presented as estimates with 95% CI, considering baseline (time point) as the reference category for change over time, considering obesity and endline (time point) as the reference categories for group BMI and group-time interaction, considering “yes” as the reference category for antihypertensive and lipid-lowering drugs, and considering male sex as the reference category for HDL-C; estimates are shown to two or three decimal places based on variable scale and interpretability; % change represents the unadjusted median percentage change from baseline to endline within each group and is provided to enhance clinical interpretability. Model effects (estimate, 95% CI, and *p* value) reflect adjusted comparisons derived from the linear mixed model. *p* value: significance of difference between baseline and endline for the respective group; all the significant values are presented in bold.

Abbreviations: BMI, body mass index; BP, blood pressure; HbA1c, glycated hemoglobin; HDL-C, high-density lipoprotein cholesterol; HOMA-B, homeostatic model assessment of beta-cell function; HOMA-IR, homeostatic model assessment of insulin resistance; LDL-C, low-density lipoprotein cholesterol; QUICKI, quantitative insulin sensitivity check index.

## Data Availability

Data are available upon reasonable request from the corresponding author.

## Nomenclature

AOR: adjusted odds ratio
BMI: body mass index
BP: blood pressure
CBT: cognitive behavior therapy
FBG: fasting blood glucose
HDL-C: high-density lipoprotein cholesterol
HOMA-B: homeostatic model assessment of beta-cell function
HOMA-IR: homeostatic model assessment of insulin resistance
IFG: impaired fasting glucose
IGT: impaired glucose tolerance
ILI: intensive lifestyle intervention
LDL-C: low-density lipoprotein cholesterol
NLP: neurolinguistic programming
Non-HDL-C: non-high-density lipoprotein cholesterol
OR: odds ratio
oGTT: oral glucose tolerance test
PP-BG: postprandial blood glucose
QUICKI: quantitative insulin sensitivity check index
REBT: rational emotive behavior therapy
WHO: World Health Organization

## Supporting Information

Supporting Information 1 Table S1: Change in lipid profile based on sensitivity analysis (*N* = 264).

Supporting Information 2 STROBE checklist.
