# Supplementary material for: Retrospective Analysis of the Efficacy of Integrated Lifestyle Modifications in Managing Prediabetes in the Indian Population
Source: J Diabetes Res. 2025 Aug 13;2025:6172692. doi: 10.1155/jdr/6172692 (PMC12367371; doi:10.1155/jdr/6172692)
Supplement: Supporting Information 1 — Table S1: Change in lipid profile based on sensitivity analysis (N = 264). [file 6172692.f1.docx]

**Table S1. Change in lipid profile based on sensitivity analysis (N=264)**

| Parameters | Baseline | | Endline | P values |
| --- | --- | --- | --- | --- |
| Total- Cholesterol (mmol/L) | 4.6 (4.0 – 5.1) | | 4.5 (4.0 – 4.9) | 0.032 |
| Triglycerides (mmol/L) | 1.2 (0.9 – 1.6) | | 1.0 (0.8 – 1.3) | <0.001 |
| LDL-C (mmol/L) | 2.9 (2.5– 3.4) | 2.8 (2.4 – 3.2) | | 0.005 |
| Non-HDL-C (mmol/L) | 3.5 (2.9 – 4.0) | | 3.3 (2.8 – 3.8) | <0.001 |
| HDL-C (mmol/L) | 1.1 (0.9 – 1.2) | | 1.2 (1.0 – 1.4) | <0.001 |
| Prevalences |  | |  |  |
| Total-Cholesterol (≥ 5.17 mmol/L) | 27.3% | | 20.1% | 0.013 |
| Triglycerides (≥1.7 mmol/L) | 22.3% | | 12.5% | <0.001 |
| LDL-C ((≥ 2.6 mmol/L) | 69.7 | | 69.7 | 1.000 |
| Non-HDL-C (≥3.36 mmol/L) | 55.3% | | 47.0% | 0.010 |
| HDL-C  (Male ≤ 1.02 mmol/L) | 45.8% | | 35.4% | 0.063 |
| (Female ≤ 1.3 mmol/L) | 66.1% | | 58.3% | 0.074 |

*Data are presented as medians (interquartile ranges) and percentages. Baseline and endline measurements were compared using the Wilcoxon signed-rank and McNemar’s tests, LDL-C: Low-density lipoprotein cholesterol; HDL-C: High-density lipoprotein cholesterol; Non-HDL-C: Non-high-density lipoprotein cholesterol; BP: Blood pressure; Non-HDL-C =Total cholesterol minus HDL-C*
